# Antioxidant Effects of *Euterpe Oleracea Mart.* (Açai) on Myocardial Ischemia-Reperfusion Injury in Rats: Would it Represent a Good Way To Follow?

**DOI:** 10.36660/abc.20190770

**Published:** 2020-01

**Authors:** Allan Kardec Nogueira de Alencar

**Affiliations:** 1 Faculdade de Medicina de Petrópolis (FMP), Petrópolis, RJ - Brazil

**Keywords:** Euterpe Olerácea, Oxidative Stress, Energy Metabolism, Myocardial Ischemia, Myocardial Reperfusion Injury, Rats

Abrupt occlusion of an epicardial coronary artery may result in acute myocardial infarction with elevation of the ST segment as the myocardium underwent an ischemic process.^1^ This promotes damage to the cardiomyocytes, mainly due to metabolic disturbances in the ATP generation with subsequent cell death and myocardial necrosis. Furthermore, reduced levels of intracellular ATP leads to an overload of cytosolic Ca^2+^ and Na^+^ concentrations and heart function impairment.^2^ It would be expected that restoration of blood flow to the ischemic area in the myocardium to minimize the injury, but reperfusion may also induce additional damage to the cardiac cells, a phenomenon described as ischemia-reperfusion (I/R) injury^3^ which contributes to an increased infarct area and microvascular dysfunction, being sometimes lethal.^4^ Myocardial I/R injury involves several features that potentiate the final damage on the heart. Morphologically, I/R lesions presents contraction bands, karyolysis, disturbance of mitochondria, sarcolemma disruption, microvascular destruction, interstitial hemorrhage, and inflammation.^5^ Additionally, at reperfusion period an elevation in the production of reactive oxygen species (ROS) displays important roles for the I/R injury extent.^6^ The respiratory chain and NADPH oxidases of the NOX family are major sources of ROS that trigger the opening of mitochondrial permeability pore, causing irreversible damage to the cardiomyocytes.^6^ Usually, the rupture of atheroma and partial or complete obstruction of an epicardial coronary artery is followed by spontaneous or interventional reperfusion. However, reperfusion sometimes may not occur.^7^ Thus, we might realize that these burdens of factors augment the final infarct size and that it is complicated to recapitulate them by using animal models considering the vast anatomic and physiological differences from the human scenario. Nevertheless, most of the current knowledge about I/R-induced myocardial damage is derived from experimental studies in animals^5^ and rodent models of I/R injury can help to clarify potential pathophysiological mechanisms and identify new targets to treat this clinical condition. In this context, rat models of myocardial infarct and I/R injury have been instigating the pre-clinical research field to establish the therapeutic potential of natural agents in innumerous diseases. The antioxidant effect observed in some plant components might be useful for proposing their applicability to induce cardioprotection, and this characteristic could be, in part, proven by administrating natural products, for example, in rats with experimentally induced cardiac injury.

*Euterpe oleracea* Mart., popularly known as “açai,” is a fruit extensively cultivated in the North region of Brazil, specifically in the Amazon. This purple fruit was chemically studied, and it was found several antioxidant substances on its composition.^8,9^ Additionally, açai has anti-inflammatory and vasodilator effects.^9,10^

A recent work has been performed by Alegre et al.,^11^ in which the authors address that açai supplementation prevents metabolism deregulation in an acute rat model of myocardial I/R injury. This study shows that preventive treatment with açai attenuates oxidative stress but did not decrease the infarcted area or improve left ventricular function after global I/R. The authors described a beneficial effect of açai only in the metabolism of heart cells and discuss that the reduction of oxidative stress would be followed by an improvement in the left ventricular function. However, they observed that the treatment with açai worsened diastolic function after I/R leading to infer that the left heart dysfunction depends on mechanisms other than oxidative damage. Additionally, despite the changes observed in antioxidant enzymes in this work, açai supplementation did not influence the expression of transcriptional factor NF-kB, Nrf2, SIRT1, and FOXO1, proteins related with oxidative stress, regulation of the antioxidant enzymes production and cellular balance by acting on apoptosis, mitochondrial biogenesis, inflammation, glucose and lipid metabolism. I understand that this data can be justified by the protocol used in this work, as probably there was not enough time for the I/R injury to stimulate protein transcription. But, in my opinion, the preventive treatment with açai for six weeks, should have avoided, at least partially, the higher levels of some of these markers after global I/R injury in hearts from male rats, as it acts as an antioxidant agent.

It is also addressed that açai supplementation led to a higher activity of b-hydroxyacyl-CoA dehydrogenase and citrate synthase enzymes which can characterize higher fatty acid oxidation. Moreover, there was a lower activity of phosphofructokinase, the enzyme for glycolysis.^11^ The authors explain that açai supplementation altered substrate selection for mitochondrial oxidation in reperfusion from glucose to fatty acids, maintaining the energy metabolism closer to a physiological situation. In this regard, I perceived a contradictory discussion of the presented results as the authors defend that açai treatment could be beneficial by altering ATP production from glycolysis to fatty acid oxidation, but later in the discussion they assume that the change in energetic metabolism which occurs in stress situations has a protective role in the myocardium and that the prevention of glucose use induced by açai supplementation may have negatively interfered with this adaptive protective mechanism.

Finally, authors comment that the amount of açai ingested by rats on their study is equivalent to 600 mg for a 60 kg human, assuming that through the data obtained in this work the quantity of açai is feasible for human ingestion, and inaccurately extrapolate their finds when, by the end of discussion, it is highlighted that açai could be a potential strategy to attenuate I/R injury in the clinical setting. How could the dose of açai administered in rats with a cardiac lesion which promoted worsening of the diastolic function be suitable for human use? This was superficially discussed by the authors when they mentioned findings from a previous work that investigated the effects of anthocyanin extract in global rat heart I/R, suggesting that this substance, which is present in the açai composition, was cardioprotective in low doses and could be cardiotoxic in high doses.^12^ Howbeit, this topic of the discussion was not clear on how this argument could justify the effects of administration of a standard chow supplemented with 5% açai in rats for six weeks, as it was not measured the anthocyanin content, the treatment with açai did not promote protection against I/R injury and the authors still remained proposing that the dose of açai used in their experimental protocol could be used in humans.

Relevant points must be considered when pre-clinical experiments are developed. This was well discussed by Ibáñez et al.,^5^ when they address the importance of comparing results from different models of I/R or even different laboratories. The time of the day at which the cardiac I/R injury is induced has a significative influence on the tolerance of the heart to that lesion.^5^ Moreover, the season and day of the week may influence the results observed in animal models and in the response of possible new cardioprotective therapies.^5^ Importantly, this is also observed in patients, as the circadian clock influences a number of cardiovascular pathophysiological processes including the incidence of acute myocardial infarction.^13^

Natural products (herbs) have in numerous substances on their composition. These compounds would interact with multiple biological targets. Thus, it is of utmost importance to identify the associations among bioactive components of herbs and their targets in the cells. Many herbal substances which are currently used have not been submitted to minacious scientific evaluations, and this might promote potential and serious toxic effects due to possible drug-to-drug interactions and/or related to the dose administrated.

Despite the important progress in pre-clinical and clinical trials evaluations of novel cardioprotective agents, it is well recognized by the cardiology field that there is a huge challenge in the development of new drugs against I/R injury, which is to perform larger phase III trials in order to elucidate clinical responses to these new substances in the context of lethal I/R. Thus, I agree that it would be worth the effort of polishing the already available therapeutic strategies instead of trying to identify new treatments for myocardial damage promoted after an episode of I/R.
